# Elicitation of Antimicrobial Active Compounds by *Streptomyces*-Fungus Co-Cultures

**DOI:** 10.3390/microorganisms9010178

**Published:** 2021-01-15

**Authors:** Matthieu Nicault, Ali Zaiter, Stéphane Dumarcay, Patrick Chaimbault, Eric Gelhaye, Pierre Leblond, Cyril Bontemps

**Affiliations:** 1 Université de Lorraine, INRAE, DynAMic, F-54000 Nancy, France; matthieu.nicault@univ-lorraine.fr; 2 Université de Lorraine, INRAE, IAM, F-54000 Nancy, France; eric.gelhaye@univ-lorraine.fr; 3 Université de Lorraine, LCP-A2MC, F-57000 Metz, France; ali.zaiter@univ-lorraine.fr (A.Z.); patrick.chaimbault@univ-lorraine.fr (P.C.); 4 Université de Lorraine, INRAE, LERMAB, F-54000 Nancy, France; stephane.dumarcay@univ-lorraine.fr

**Keywords:** *Streptomyces*, fungus, co-culture, specialized metabolism, biosynthetic gene cluster, antimicrobial agents

## Abstract

The bacteria of the genus *Streptomyces* and Basidiomycete fungi harbor many biosynthetic gene clusters (BGCs) that are at the origin of many bioactive molecules with medical or industrial interests. Nevertheless, most BGCs do not express in standard lab growth conditions, preventing the full metabolic potential of these organisms from being exploited. Because it generates biotic cues encountered during natural growth conditions, co-culture is a means to elicit such cryptic compounds. In this study, we explored 72 different *Streptomyces*-fungus interaction zones (SFIZs) generated during the co-culture of eight *Streptomyces* and nine fungi. Two SFIZs were selected because they showed an elicitation of anti-bacterial activity compared to mono-cultures. The study of these SFIZs showed that co-culture had a strong impact on the metabolic expression of each partner and enabled the expression of specific compounds. These results show that mimicking the biotic interactions present in this ecological niche is a promising avenue of research to explore the metabolic capacities of *Streptomyces* and fungi.

## 1. Introduction

Microorganisms form complex multispecies communities in all environments (e.g., soil, oceans, microbiota) where they play key roles. In soil, they contribute to homeostasis by participating in biogeochemical cycles [1] or by improving nutrient availability for plants [2]. Bacteria and fungi of a same community are in constant interaction (commensalism, mutualism, competition or antagonism) that can affect their growth or induce more specific behaviors, such as pathogenicity [3]. These bacterial-fungal interactions (BFIs) are often driven by the production of specialized metabolites (SMs). The latter can have a direct impact (e.g., antibiosis, sugar degradation) but can also act as communication signals and trigger expression of other specific biosynthetic pathways in other niche inhabitants [4]. Among these SMs, some exhibit interesting biological properties (e.g., antimicrobials) and represent a source for the conception of new drugs [5].

In that respect, fungi and bacteria of the genus *Streptomyces* are of special interest, as they provide alone 42% and 32% of the natural products used in the human and veterinary medicine, respectively [6]. For *Streptomyces*, genomic analyses have shown that they possess between 20 and 50 biosynthesis gene clusters (BGCs) predicted to produce SMs [7,8]. Yet, *Streptomyces* strains are generally used for the production of a single or a few compounds. For example, *Streptomyces griseus*, the producer of the antibiotic streptomycin [9] and *Streptomyces avermitilis*, the producer of the polyketide anthelmintic avermectin [10] possess 34 and 25 BGCs, respectively [11,12]. The genetic model *Streptomyces coelicolor* A3(2), for its part, was known for a long time to encode only three antibiotics (the aromatic polyketide antibiotic actinorhodin, oligopyrrole prodiginine antibiotics, and the non-ribosomal peptide calcium-dependent antibiotic) [13,14], although its genome contains 20 BGCs [13]. The access to the genome sequences of many other species since has confirmed that the potential to code for specialized metabolites far exceeds the activities and compounds that can be identified based on bioassays or the detection of direct molecules, and that most BGCs remain silent in standard laboratory conditions [15].

It is therefore important to find new methods to explore and to induce the production of specialized metabolites by activating these biosynthetic pathways [16,17], and many methods were developed for that purpose over the past decades. For instance, it has been successfully achieved by rewiring regulatory networks [18], ribosome engineering, regulatory unlocking, heterologous expression [19,20,21], or by randomly modifying parameters of culture conditions (e.g., nutrient source, culture medium) [22]. In recent years, there has been a renewal of interest to express cryptic SMs in the lab through co-culture approaches [23]. Biotic stresses are indeed likely to trigger adaptive responses (e.g., competitive weapons, hormones, communication molecules) by mimicking signals emitted by community co-inhabitants [24].

Co-cultures were described with various bacteria and fungi [23]. Regarding fungi, wood-decaying fungi have already been used [25,26] and are of special interest as they are known to control and to modify bacterial communities in their environment [27,28], in particular through metabolites production and pH modification [29]. They thus constitute good candidates to produce antibacterial compounds or to trigger such production in interaction.

For bacteria, as *Streptomyces* remain one of the main source of SMs, they were often co-cultivated together [30,31] or with other bacterial genera [32,33,34,35]. *Streptomyces*-fungi co-cultures are on the other hand rarer, despite having showed promising results. For instance, co-cultures of *Aspergillus fumigatus* MR2012 with *Streptomyces leeuwenhoekii* C34 [36] or *Streptomyces rapamycinicus* and the model fungus *Aspergillus nidulans* [37] led to the production of new compounds.

The aim of our work was to exploit a *Streptomyces* strain collection isolated from the same soil microhabitat in co-cultures with different wood-decaying fungi in order to stimulate the production of antibacterial compounds. Bi-partite co-cultures were set up and the antimicrobial activity was assessed against indicator bacterial strains (mostly Bacilli). Monitoring the antimicrobial activity was used as a screening method throughout the analytical processes. Here we observed an important induced chemical diversity that explains some mechanisms of microbial interactions. Activity-guided separation led to the detection of candidate compounds that could be responsible for the induced antimicrobial activities.

## 2. Results and Discussion

### 2.1. Induction of Antibacterial Activities by Streptomyces-Fungus Co-Culture

**Figure 1:**
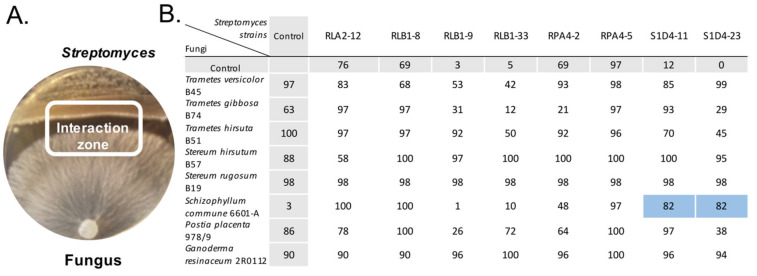
*Streptomyces*-fungus co-culture setup and screening. (**A**) Illustration of a *Streptomyces*-fungus co-culture after 14 days of growth. (**B**) Screening of bioactive molecules elicited during the co-culture. The compound extracts of mono-cultures of fungi and *Streptomyces* (rows and columns named “control”) in GA medium were compared with the extracts resulting from their co-culture in a bioassay experiment against the growth of *B. subtilis* ATCC6633. The values in the table indicate the percentage of inhibition of *B. subtilis* ATCC6633 after a 24 h growth period in comparison with a control grown in absence of the extract. The two co-cultures between *S. commune* 6601-A with S1D4-11 and S1D4-23 (highlighted in blue) were selected as they presented a significant (*t*-test *p* value < 0.05) impact on the growth of *B. subtilis* ATCC6633 in comparison with the controls.

To set up bacterial–fungal co-cultures, we used two collections of the soil dwelling bacterium *Streptomyces* and of forest basidiomycetes available in our laboratories (Figure 1). On the one hand, the bacterial collection was made up of eight *Streptomyces* isolated from the same forest soil micro-habitats and belonging to identical or different species as previously described by Nicault et al. [8]. Their genome sequences were determined and showed a great diversity of their specialized metabolite BGCs, with 261 identified for the eight strains [8]. On the other hand, the fungal collection was made up of wood-decaying basidiomycetes. Besides the well-known ability of wood-decaying fungi to control bacterial communities [38,39], the fungi have been chosen for their potential to produce antimicrobial products [40,41,42,43,44], for instance the genera *Trametes* [45] or *Stereum* [46]. In our experimental design, bacterial and fungal couples were grown on agar plates at each side of the petri dishes (Figure 1A) generating a *Streptomyces*-fungus interaction zone (SFIZ). A similar design was previously used to reveal compounds induced during co-cultures, for instance with marine fungi [47]. Our case had the advantage to enable the interaction either by a diffusible signal in the agar or through contact between the two partners—two interaction types already described in *Streptomyces* and fungal co-cultures [48,49]. As nutritive sources can have a profound effect on specialized metabolism expression, we only used the GA medium that is commonly used for *Streptomyces* culture. Our initial collection consisted of eight *Streptomyces* and nine fungi and all of them with the exception of *Phaeolus schweinitzii* were able to grow on GA medium, resulting in 64 different co-cultures (Figure 1B). In order to survey the impact of the co-culture on the metabolome, we focused on metabolites that were expressed specifically through co-culture, that is to say those that remained cryptic in mono-cultures. For that purpose, we tested the inhibition activity of the ethyl acetate extract of the inhibition zone in comparison to the mono-culture controls against the indicator *Bacillus subtilis* ATCC6633 strain (Figure 1B). Each interaction was screened once and only experiments showing an elicitation by co-culture of the anti-*Bacillus* activity were repeated five times. Two out of 64 interactions revealed co-culture elicitations (Figure 1B). This number is in the same range as reported by Schroeck et al. [37], where elicitation was found in one interaction out of 58 between *Aspergillus nidulans* and different *Streptomyces*. In our case, most controls, either bacterial or fungal, showed activity against *B. subtilis* ATCC6633 and prevented the revealing of a potential elicitation during the screening. This result confirms that fungi and *Streptomyces* are prominent sources of bioactive molecules and we can speculate that the number of elicitations is probably underestimated here.

The two selected couples were both composed of the fungus *S. commune* 66-01A with *Streptomyces* S1D4-11 or S1D4-23. *S. commune* is a wood-decaying fungus that can induce melanin, indole, flavonoids, and carotenoids when grown in the presence of co-occurring bacteria or fungi [50]. It also represents a good interaction model with *Streptomyces*, as it was shown to restore hyphal formation-deficient phenotypes in some strains [51]. S1D4-11 and S1D4-23 are closely related but different species of *Streptomyces* and each were previously predicted to harbor 36 BGCs with 25 in common and 11 specific ones [8]. It can be noticed that the two other *Streptomyces* RLB1-33 and RLB1-9 that showed no anti-*B. subtilis* activity in mono-culture (Figure 1B) had no induced capacity in their SFI zones with *S. commune*. This result highlights that whether the elicited activity came from the fungus or the bacteria, it was specific between *S. commune* and the strains S1D4-11 and S1D4-23.

**Figure 2:**
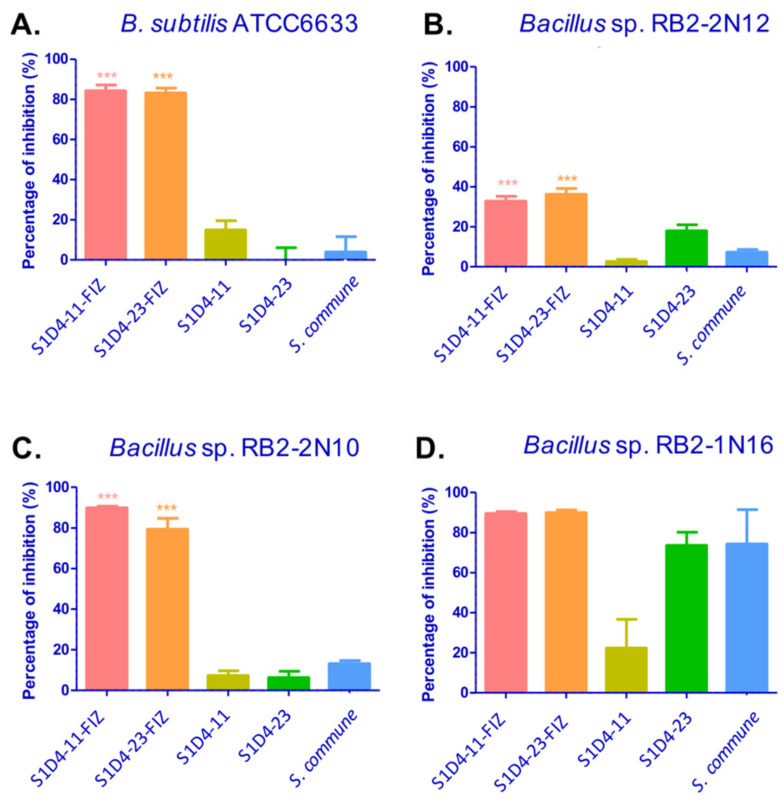
Antimicrobial activities of S1D4-11-FIZ and S1D4-23-FIZ against different *Bacilli*. Inhibition was quantified as a percentage of growth inhibition in comparison with a control without extract. The growth was measured by spectrometry at OD 600 nm after 24 h of growth. The different Bacilli strains are (**A**) *Bacillus subtilis* ATCC6633, (**B**) *Bacillus* sp. RB2-2 N12, (**C**) *Bacillus* sp. RB2-2 N10, and (**D**) *Bacillus* sp. RB2-1 N16. Statistical difference was assessed with a *t*-test. *** = *p* value < 0.005 for both comparisons between fungus and *Streptomyces*-fungus interaction zone (SFIZ) and *Streptomyces* and SFIZ.

The interactions with elicitation against *B. subtilis* ATCC6633 were named S1D4-11-FIZ and S1D4-23-FIZ. They were tested against three additional environmental *Bacillus* strains, isolated from the same soil as the *Streptomyces* collection (Figure 2), and strains RB2-2N12 and RB2-2N10 appeared sensitive to the SFIZ extracts though at different levels in comparison with the controls. Antibacterial activity of S1D4-11-FIZ and S1D4-23-FIZ extracts were also tested against other indicator strains: *Staphylococcus aureus* ATCC29213, *Escherichia coli* ATCC25922, *Enterococcus faecalis* ATCC292212, *Pseudomonas aeruginosa* ATCC27853, *Acinetobacter baumanii* ATCC19606, *Klebsiella pneumonia* ABC42, and *Enterobacter cloacae* ABC291. No significant activity was detected against these bacterial species Although we did not perform an extensive screening and only revealed anti-*Bacillus* activities, our results show that co-culture can revealed antibacterial activities not expressed in standard lab conditions. This extended approach, when applied to other bacteria of interest, such as antibiotic multi-resistant bacteria, should provide a simple and generalizable means of discovering new biomolecules.

### 2.2. Impact of Co-Culture on Metabolome

**Figure 3:**
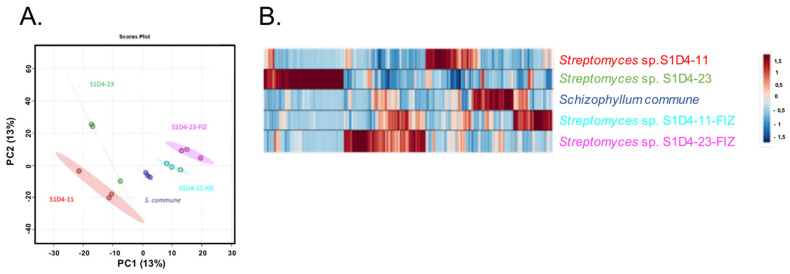
Gas chromatography-mass spectrometry (GC-MS) spectrum comparison. (**A**) Partial least square-discriminant analysis (PLS-DA)comparison of GC-MS spectra of mono- and co-cultures. (**B**) Heat-map of the first 750 discriminant features (26%) revealed by PLS-DA. The scale indicates the relative abundance of features calculated by centered-reduced of initial intensity.

**Figure 4:**
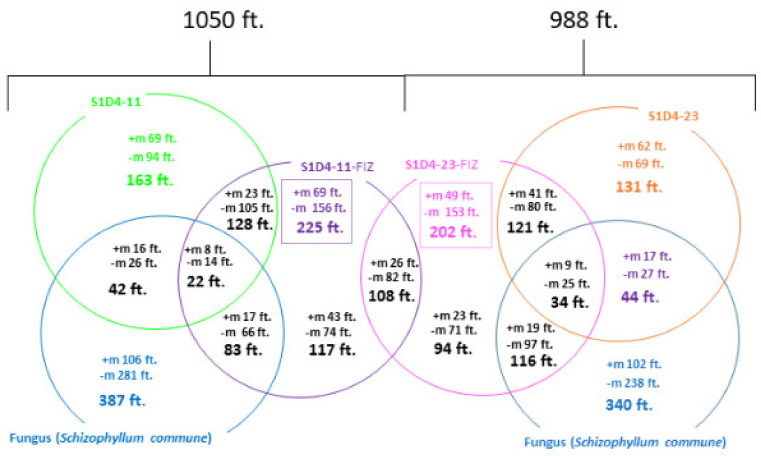
Distribution of common and specific features between SFIZs and controls. LC-MS metabolite profiles were recorded and analyzed with GNPS. The Venn diagram compares specific and common features between each SFIZ and its controls as well as SFIZ features between the two experiments. The number of SFIZ specific features for each experiment is indicated in the dashed square. +m: positive mode; −m: negative mode; ft.: features.

In order to decipher the metabolic impact of co-culture, molecular profilings of the two selected SFIZs as well as the corresponding pure culture extracts were analyzed by gas chromatography coupled to a mass spectrometer (GC-MS). GC-MS was already used for the characterization of bioactive *Streptomyces* strains [52] and the used derivatization enabled us to detect more compounds, such as amino acids and lipids. Based on ions extracted from mass spectra, 2796 features were detected with MzMine 2 software and partial least square-discriminant analysis (PLS-DA) showed that each extract (controls and SFIZs) can be discriminated from the others (Figure 3A). From the PLS-DA, discriminating variables (26%) were compared in a heatmap (Figure 3B) and only showed slight overlap, giving a unique bar code for each condition. These results show that mono-culture extracts are different and that each microbe has a specific metabolomic signature. Secondly, the extracts from the co-cultures differed from their controls, indicating that a specific production of compounds was induced during the interaction. To confirm these results with more polar and soluble compounds [53], SFIZ and control extracts were analyzed by ultra-high-performance liquid chromatography with tandem mass spectrometry (LC-MS/MS). In the co-culture experiment between S1D4-11 and *S. schizophyllum*, 387 features were specific to the fungus extract (i.e., not found in other extracts) and 163 to the bacterial extract (Figure 4). In the co-culture between S1D4-23 and *S. schizophyllum*, 340 features were specific to the fungus extract and 131 to the bacterial extract. Regarding the SFIZs of these two co-cultures, 458 and 473 features were respectively identified, with around a quarter being shared with either the fungal or the bacterial controls, logically reflecting that they are produced during both mono- and co-cultures. Interestingly, a greater proportion of these SFIZ features were specific (225 for S1D4-11-FIZ and 202 for S1D4-23-FIZ) and confirmed that co-cultures greatly affect the metabolome. Moreover, almost half of these SFIZ specific features (*n* = 108) were shared between S1D4-11-FIZ and S1D4-23-FIZ, suggesting that a significant part of the specific metabolic response induced by the interaction is common between the two co-cultures. We can speculate that most of these shared compounds are produced by *S. commune,* which is common to both co-cultures, and/or by a common metabolism induced in *Streptomyces* in response to the fungus.

### 2.3. Towards the Identification of Potential Bioactive Compounds

**Figure 5:**
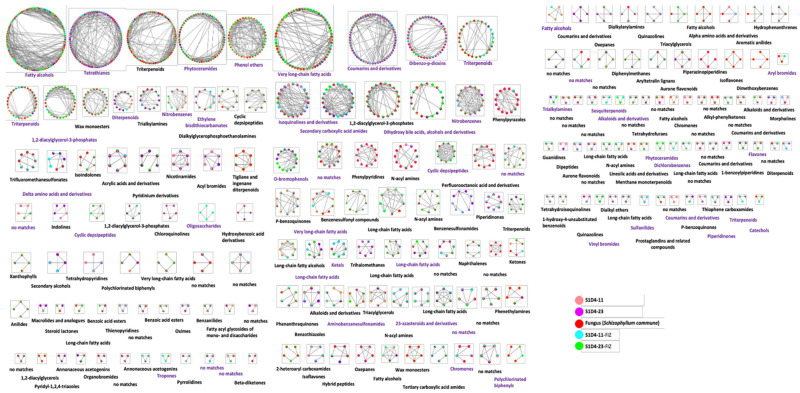
Molecular networking of ions produced during mono- and co-cultures. Data were obtained byLC-MS/MS in both positive and negative modes and were analyzed through GNPS. Each spectrum is shown as a node and related metabolites are linked by edges. Node color represents fractions by a chromatographic separation (C18 reverse phase) *prior* MS/MS detection. For both SFIZ extracts, the activity against the indicator strain was only found in two fractions (N° 22 and 23). To identify potential candidates induced during the studied interactions, we selected compounds found in fractions 22 and 23 and in SFIZs but not in the controls (Figure 6). As control extracts were solubilized in dimethyl sulfoxid (DMSO) (see previous paragraph), we restricted this analysis to SFIZ extract molecules found in both DMSO and methanol, recognizing that some methanol-soluble and DMSO-insoluble candidates could be omitted.

To identify the chemical actors of these specific metabolic responses, a LC-MS molecular networking with GNPS (Global Natural Products Social Molecular Networking) that gathers MS/MS spectra into networks corresponding to chemical families was performed. In total, 187 networks were detected with 159 identified as chemical families (Figure 5). The non-identified molecule families (labeled “no matches”) could correspond to new molecules, and half of SFIZ compounds (nodes within networks) were present in this category. It might suggest that the co-cultures induced a less characterized metabolism than single cultures that can be the source of innovative molecules. Four networks were found to be specific to one or both SFIZs. Among them, S1D4-11-FIZ presented specific oligosaccharides, S1D4-23-FIZ presented phytoceramides and vinyl bromides, and both SFIZs had specific alkaloids and derivative compounds that can have pharmaceutical properties, including antibacterial activities [54].

Although the mass spectrometer used in this work has a low resolution that did not allow identifying compounds of interest through the determination of molecular formulae, the LC-MS and LC-MS/MS profiles enabled us to compare samples obtained in the different conditions of culture in terms of the presence and absence of some specific metabolites. Hence, for the four fractions, one specific peak was identified in fraction 22 of S1D4-11-SFIZ, five in fraction 23 of S1D4-11-SFIZ, five in fraction 22 of S1D4-23-SFIZ and 21 in fraction 23 of S1D4-23-FIZ. For each couple of co-cultures, no MS peak was common between the two fractions, indicating that different molecules are responsible for the anti-*Bacillus* inhibition activity, or that their concentration is too low in a fraction compared to the others detected by MS. Determining which partner produces these different compounds was not possible, but it can be noticed that a few peaks were common between the two SFIZs. As the fungal partner is the same in both co-cultures, this suggests that most of the compounds were produced by variable gene pools in *Streptomyces* or that the fungal response is different and specific depending on its partner.

Characterizing the molecule(s) responsible for the activity is undoubtedly the next challenge. Obtaining higher precision on the measure of the m/z values using as a minimum a Q-TOF (Time of Flight), but even better an Orbitrap or FTICR mass spectrometer will allow the determination of molecular formulae of intact molecules and their fragments facilitating their characterization using local MS databases and/or the literature. If no matching is obtained from these new data, this could also mean that these compounds belong to the dark metabolome, whose exploration is promising for the discovery of new antibiotics. In this case, genome analysis coupled with genetics approaches will be used to target and knock-out specific BGCs previously identified [8]. The loss of activity in a mutant will enable identifying the bioactive molecules and decipher the black metabolome of our *Streptomyces*-fungus interactions.

## 3. Conclusions

Taken together, these results suggest that co-culture had a significant effect on the metabolic expression of both organisms and that the metabolome of the interaction is not the simple addition of compounds produced by the fungi and those of the bacteria. In order to best explore this important diversity of metabolites, we have chosen an approach guided by anti-*Bacillus* inhibition activity induced by co-culture, and tried to link metabolome and phenotype. Interestingly, even if the precise identification of one or several metabolites could not be fully achieved, we were able to show that bioactive fractions had specific molecules, which are potentially antimicrobial candidates. Our results confirm that co-culture coupled with an activity-targeted approach represents an excellent means of activating cryptic metabolites in the laboratory and could lead to the discovery of molecules of interest.

## 4. Materials and Methods

### 4.1. Strains and Culture Conditions

The bacterial and fungal strains used in this work are listed in Figure 1. The *Streptomyces* strains were stored at −20 °C as spore suspensions in glycerol 20% and were grown on GA medium (15 g starch, 5 g NaCl, 5 g KCl, 0.5 g K_2_HPO_4_, 0.5 g KNO_3_, 0.5 g MgSO_4_, 15 mg FeSO_4_, and 15 g Bacto agar in 1 L of water adjusted to pH 7.2). *Bacillus* strains were grown in lysogeny broth (LB) (10 g tryptone, 5 g yeast extract, 10 g NaCl for 1 L) at 30 °C. Fungi were maintained on MA medium (15 g malt extract and 15 g Bacto agar) by successive cultures. Co-cultures were performed in triplicate on GA medium plates. On one side of the Petri dish, an agar plug with fresh fungal mycelium was inoculated and 10 µL of *Streptomyces* spore suspension (10^5^ spores/mL) were streaked on the opposite side (on approximately one third of the Petri dish, as illustrated in Figure 1A). The co-cultures were grown for 10 to 14 days at 25 °C in the dark. Controls were performed similarly but only with one microorganism.

### 4.2. Metabolite Extraction

Interaction zones (Figure 1A) from three independent Petri dishes were excised and pooled together in a flask with one volume of ethyl acetate (99.8%, Biosolve, Dieuze, France). After 90 min of agitation (120 rpm) at room temperature, ethyl acetate extracts were recovered by filtering on a paper filter of 100 µm and were vacuum-dried at room temperature. Dry extracts were resuspended at a concentration of 10 mg/mL either in DMSO (extra dry, 99.8%, Biosolve) or in methanol (HPLC quality, 99.9%, Carlo Erba, Val de Reuil, France). For fractioning experiments, 80 Petri dishes were pooled to obtain the final extract.

### 4.3. Anti-Bacterial Screening

Anti-bacterial screening tests were performed in 96-well plates by adding 5 µL of extract to a 200 µL culture (0.05 OD 600 nm) of the *Bacillus* indicator strain. Culture growth was monitored every 10 min for 24 h (30 °C, rapid shake mode) on a BioTech (Synergy HT, Winooski, U.S.A). The initial 64 different co-culture extracts were screened once against *B. subtilis* ATCC6633 and those with activity, as well as controls, were repeated at least five times independently. Antibacterial assays against other bacterial species (*Staphylococcus aureus* ATCC29213, *Escherichia coli* ATCC 25922, *Enterococcus faecalis* ATCC 292212, *Pseudomonas aeruginosa* ATCC 27853, *Acinetobacter baumanii* ATCC 19606, *Klebsiella pneumonia* ABC 42, and *Enterococcus cloacae* ABC 291) were performed on the ABC Platform (Université de Lorraine, Vandoeuvre-Lès-Nancy, France) according to the ISO 20776-1 protocol.

### 4.4. GC-MS and LC-MS/MS Metabolite Profiling

Dried extracts were silylated by adding 50 μL of BSTFA/TMSCl (99/1) and heated at 50 °C for 12 h. After evaporation of the derivatizing reagent, and the extract derivatives were solubilized in ethyl acetate and transferred for gas chromatography analysis. GC-MS analysis was performed on a Clarus 680 gas chromatograph coupled to a Clarus SQ8 quadrupole mass spectrometer (Perkin Elmer Inc., Waltham, MA, USA). Gas chromatography was carried out on a 5% diphenyl/95% dimethyl polysiloxane fused-silica capillary column (DB-5 ms, 30 m × 0.25 mm, 0.25 μm film thickness, J&W Scientific, Folsom, CA, USA) with helium as a carrier gas at a constant flow of 1 mL/min. The gas chromatograph was equipped with an electronically controlled split/splitless injection port. The injection (1 μL) was performed at 250 °C in the splitless mode. The oven temperature program was as follows: 80 °C for 2 min, increase from 80 to 190 °C at a rate of 10 °C/min, increase from 190 to 280 °C at a rate of 15 °C/min and hold for 5 min, then 10 °C/min until 300 °C hold for 14 min. Ionization was achieved under the electron impact mode (70 eV ionization energy). The source and transfer line temperatures were 180 and 250 °C, respectively. Detection was carried out in scan mode: m/z 45 to m/z 700. The detector was switched off in the initial 2 min (solvent delay).

LC-MS/MS was performed with a Thermo Scientific ultimate 3000 liquid chromatography with a mass detector IonTrap LTQ Velos pro (Thermo Scientific, San Jose, CA, USA) fitted with an ESI source (capillary and source temperatures are 300 and 250 °C, respectively). Detection was carried out in positive (+5 kV) and in negative (−4 kV) ion mode from m/z 100 to m/z 2000. The five most intense peaks in the MS spectra were submitted to fragmentation by CID at 45 eV-collision energy.

Separation was carried out with a C18 HypersiI gold aQ column (100 × 2.1 mm ID, 5µ particle, Thermo Fischer Scientific, Waltham, MA, USA). Analysis was performed in gradient mode at a flow rate of 200 µL/min. The column was equilibrated during 20 min with 10% methanol, 5% acetonitrile and 85% water. After the sample injection (5 µL), the gradient started from 10% to 50% of methanol, and from 5% to 50% of acetonitrile over 30 min.

### 4.5. Extract Fractioning

Fractioning was performed with an HPLC Shimadzu LC-20AT coupled with a DAD SPD-20A detector. The semi-preparative column was in a Kinetex biphenyl stationary phase (300 mm * 4.6 mm, 5µ particle of 100 A, Phenomenex, Torrance, CA, USA). Analysis was performed in gradient mode at a flow rate of 1 mL/min. The gradient started from 0% to 100% methanol during 55 min. The column was washed for 25 min with 100% water. All fractioning were realized four times with 100 µL of 10 mg/mL extracts.

### 4.6. Molecular Networking

A molecular network was created using the online workflow (https://ccms-ucsd.github.io/GNPSDocumentation/) on the GNPS website (http://gnps.ucsd.edu). The data were filtered by removing all MS/MS fragment ions within +/−17 Da of the precursor m/z. MS/MS spectra were window-filtered by choosing only the top six fragment ions in the +/−50 Da window throughout the spectrum. The precursor ion mass tolerance was set to 2.0 Da and a MS/MS fragment ion tolerance of 0.5 Da. A network was created where edges were filtered to have a cosine score above 0.7 and more than six matched peaks. Furthermore, edges between two nodes were kept in the network if and only if each of the nodes appeared in each other’s respective top 10 most similar nodes. Finally, the maximum size of a molecular family was set to 100, and the lowest scoring edges were removed from molecular families until the molecular family size was below this threshold. The spectra in the network were then searched against GNPS’ spectral libraries. The library spectra were filtered in the same manner as the input data. All matches kept between network spectra and library spectra were required to have a score above 0.7 and at least six matched peaks.

### 4.7. Statistical Analysis

PLS-DA and the heatmap were performed using the Metaboanalyst platform. Statistical analyses were *t*-tests and were performed with R 3.6.1 software.

## Figures and Tables

**Figure 6:**
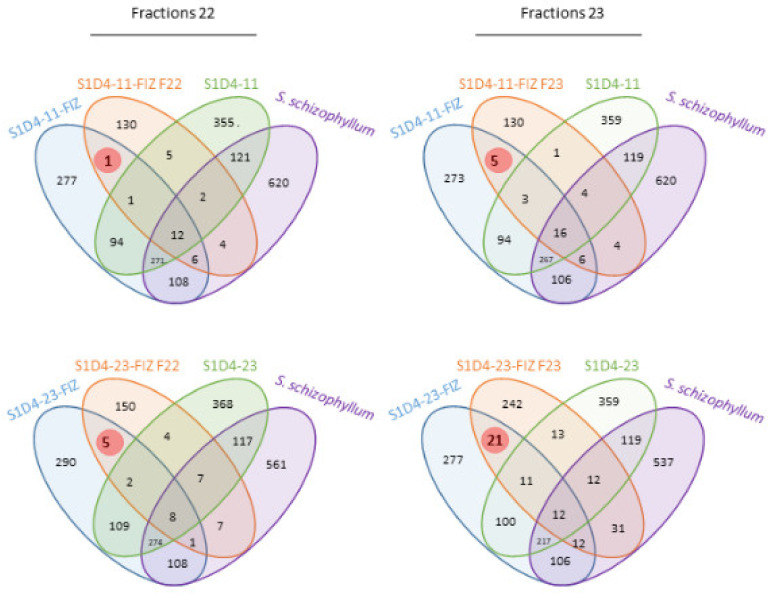
Identification of specific compounds in bioactive fractions. LC-MS spectra of fractions N° 22 and N° 23 (named S1D4-11-FIZ F22 and S1D4-23-FIZ F23, respectively) with anti-*Bacillus* activity were compared with single culture controls and with total extracts of SFIZ in DMSO, which also has similar activity. Compounds with potential activities (highlighted in red) are those at the intersection of the total SFIZ extract and in the fraction considered. The number of features found in each condition is indicated. Features were analyzed and compared with DEREPLICATOR+ in order to build the Venn diagram.

## Data Availability

The data presented in this study are available on request from the corresponding author.
